# The Shared and Distinct White Matter Networks Between Drug-Naive Patients With Obsessive-Compulsive Disorder and Schizophrenia

**DOI:** 10.3389/fnins.2019.00096

**Published:** 2019-02-21

**Authors:** Jiaolong Qin, Jing Sui, Huangjing Ni, Shuai Wang, Fuquan Zhang, Zhenhe Zhou, Lin Tian

**Affiliations:** ^1^The Key Laboratory of Intelligent Perception and Systems for High-Dimensional Information of Ministry of Education, School of Computer Science and Engineering, Nanjing University of Science and Technology, Nanjing, China; ^2^National Laboratory of Pattern Recognition, Institute of Automation, Chinese Academy of Sciences, Beijing, China; ^3^Brainnetome Center, Institute of Automation, Chinese Academy of Sciences, Beijing, China; ^4^Chinese Academy of Sciences Center for Excellence in Brain Science, Institute of Automation, Beijing, China; ^5^Smart Health Big Data Analysis and Location Services Engineering Lab of Jiangsu Province, Nanjing University of Posts and Telecommunications, Nanjing, China; ^6^The Affiliated Wuxi Mental Health Center of Nanjing Medical University, Wuxi, China; ^7^Wuxi Tongren International Rehabilitation Hospital, Wuxi, China

**Keywords:** obsessive-compulsive disorder, schizophrenia, diffusion MRI, graphical measures, putamen, network topology

## Abstract

**Background:** Obsessive-compulsive disorder (OCD) and schizophrenia (SZ) as two severe mental disorders share many clinical symptoms, and have a tight association on the psychopathological level. However, the neurobiological substrates between these two diseases remain unclear. To the best of our knowledge, no study has directly compared OCD with SZ from the perspective of white matter (WM) networks.

**Methods:** Graph theory and network-based statistic methods were applied to diffusion MRI to investigate and compare the WM topological characteristics among 29 drug-naive OCDs, 29 drug-naive SZs, and 65 demographically-matched healthy controls (NC).

**Results:** Compared to NCs, OCDs showed the alterations of nodal efficiency and strength in orbitofrontal (OFG) and middle frontal gyrus (MFG), while SZs exhibited widely-distributed abnormalities involving the OFG, MFG, fusiform gyrus, heschl gyrus, calcarine, lingual gyrus, putamen, and thalamus, and most of these regions also showed a significant difference from OCDs. Moreover, SZs had significantly fewer connections in striatum and visual/auditory cortices than OCDs. The right putamen consistently showed significant differences between both disorders on nodal characteristics and structural connectivity.

**Conclusions:** SZ and OCD present different level of anatomical impairment and some distinct topological patterns, and the former has more serious and more widespread disruptions. The significant differences between both disorders are observed in many regions involving the frontal, temporal, occipital, and subcortical regions. Particularly, putamen may serve as a potential imaging marker to distinguish these two disorders and may be the key difference in their pathological changes.

## Introduction

Although schizophrenia (SZ) and obsessive-compulsive disorder (OCD) are described as distinct disorders in contemporary psychiatry, they actually have notable symptom overlap, and a tight association on the psychopathological level (Meier et al., 2014). Schizophrenia is characterized by hallucinations, delusions, loss of initiative, and cognitive dysfunction (Kahn et al., 2015), while OCD is featured by recurrent, persistent, and intrusive thoughts typically causing distress or anxiety and repetitive behaviors aimed at reducing anxiety (Pauls et al., 2014). Over the past decades, the relationship between SZ and OCD has been attracting an increasing attention since these disorders apparently share some clinical characteristics (Scotti-Muzzi and Saide, 2017). Both disorders affect male and female equally, have prevalence rates of comparable magnitude, a chronic course, and a similar distribution of age-at-onset (Rabinowitz et al., 2006; Narayanaswamy et al., 2012). However, the neurobiological substrates and the etiological relationship between OCD and SZ remain unclear (Pauls et al., 2014; Kahn et al., 2015). To address the issue, the study evidence would be more convincing if neurobiological studies are to demonstrate a distinct difference in neurobiology rather than just the summation or superimposition of neurobiological alterations observed in each disorder separately. Hence, it is necessary to investigate the association between SZ and OCD under the same methodology and research framework.

The development of promising neuroimaging techniques (i.e., diffusion tensor image, DTI), with better spatial and temporal resolutions, will allow more accurate measurements of the neurological abnormalities in psychiatric disorders. Prior neuroimaging studies summarized that SZ and OCD shared common alterations of several crucial regions including caudate, orbitofrontal cortex (OFC), and thalamus (Gross-Isseroff et al., 2003). Moreover, a few studies directly comparing SZ with OCD have reported that both have some pathophysiological similarities such as deficit of the frontostriatal circuit, but more structural abnormalities were involved in SZ (Kim et al., 2004). In recent years, advances in the development and application of DTI and graph theory methods allow for the investigation of topological patterns of brain white matter (WM) networks *in vivo* (Sporns, 2011; Bullmore and Sporns, 2012). Many studies have used such a powerful framework to probe alterations of mental disorders including SZ and OCD (Rubinov and Bassett, 2011; Fornito et al., 2012; Qin et al., 2014; Zhong et al., 2014). Altered structural connectivity and brain network topology have been described in SZ patients (Zalesky et al., 2011; Jiang et al., 2013; Rubinov and Bullmore, 2013; Rubinov et al., 2013; van den Heuvel and Fornito, 2014; Fornito and Bullmore, 2015), e.g., equivalent small-world organization and reduced network efficiency were identified in SZ patients (van den Heuvel and Fornito, 2014). In addition, structural connectome-wide analyses also reported that disrupted sub-network within frontal-posterior regions in SZ (Zalesky et al., 2010). The number of structural network study on OCD is much less relative to SZ. Specifically, Zhong et al. (2014) first constructed structural networks based on the DTI data for OCD patients and found a decrease of nodal efficiency in frontal, parietal regions, and caudate in the patients. Reess et al. (2016) employed network-based statistic (NBS) method in the WM networks for OCD patients and revealed a single decreased structural sub-network in the patients comprising OFC, striatal, insula, and temporo-limbic regions. Notably, Crossley et al. (2014) reviewed the alterations of brain structural networks among many psychiatric disorders and proposed a “disorder specific” concept which pointed that OCD showed less disrupted hubs compared with other severe mental disorders (i.e., SZ).

To the best of our knowledge, so far, no study has directly compared OCD with SZ from the perspective of anatomical networks based on WM tracts under the same research framework. Therefore, we are motivated to use DTI tractography and graph theory approaches to investigate the topological organization of the WM network in drug-naive patients with OCD and SZ, aiming to discover the common and different patterns of WM deficits between these two patient groups. We hypothesized that WM network abnormalities will be present in both SZ and OCD, with the SZ group demonstrating more serious lesions on network topological organizations than the OCD group, and both groups showing distinct deficit patterns.

## Materials and Methods

### Participants

Participants including 29 SZs, 29 OCDs, and 65 normal controls (NCs) were recruited from the inpatient and outpatient services at The Affiliated Wuxi Mental Health Center of Nanjing Medical University, China (detailed demographic and clinical information, please see Table 1). All patients met the DSM-IV-TR criteria (Association, 2000) and they did not use anti-obsessive-compulsive or anti-psychotic drugs before the MRI scanning of this study. The severity of OCD symptoms, severity of depressive and anxious symptoms were assessed by Yale-Brown Obsessive Compulsive Scale (Y-BOCS) (Goodman et al., 1989), 24-item Hamilton Rating Scale for Depression (24-HDRS) (Hamilton, 1967), and Hamilton Anxiety Rating Scale (HARS) (Hamilton, 1959), respectively. As for SZ, the evaluation of disorder severity and psychopathology was assessed by experienced psychiatrists using Positive and Negative Syndrome Scale (PANSS) (Kay et al., 1987). For patients, the assessments of symptoms were performed in the same day of their MRI scanning. The NCs were recruited from the local community via advertisements and free of the history or current diagnosis of any psychiatric disorder. Moreover, the NCs with a family history of major psychiatric or neurological illness in their first-degree relatives were excluded. All recruited participants are right-handed Han Chinese. Participants were excluded if any of the following were present: (1) the existence of alcohol or substance abuse or dependence or concomitant major medical disorder, (2) history of intracranial pathology or brain injury or any neurological disorder, and (3) any MRI contraindications. This study was approved by the Research Ethics Review Board of Wuxi Mental Health Center, and written informed consents were obtained from all participants.

**Table 1:** Demographic information of the samples in this study.

**Variables**	**NC (*n* = 65)**	**OCD (*n* = 29)**	**SZ (*n* = 29)**	***p*-value**
Age (years)	17–50 (32.35 ± 10.73)	16–43 (26.45 ± 8.12)	16–61 (32.76 ± 10.37)	0.021^*^
Education (years)	6–23 (13.97 ± 3.66)	6–19 (13.31 ± 2.90)	0–19 (10.93 ± 4.50)	0.002^*^
Gender (M/F)	41/24	23/6	17/12	0.217^#^
Handedness (R/L/A)	65/-/-	29/-/-	29/-/-	>0.999
Disease duration (years)	–	0.5–18 (4.55 ± 4.47)	0.5–20 (3.27 ± 4.37)	0.274
PANSS positive score	–	–	19–38 (27.62 ± 4.19)	–
PANSS negative score	–	–	8–30 (20.45 ± 4.93)	–
PANSS general score	–	–	22–56 (46.83 ± 6.73)	–
PANSS total score	–	–	53–114 (94.90 ± 11.18)	–
Y-BOCS score				
Obsession score	–	4–20 (12.38 ± 3.77)	–	–
Compulsive score	–	5–15 (8.76 ± 3.00)	–	–
Total score		9–28 (22.20 ± 5.16)		
HARS score	–	4–42 (16.17 ± 7.26)	–	
24-HDRS score	–	4–31 (16.93 ± 8.05)	–	

*Data is described as the minimum-maximum (mean ± SD)*.

* p-values are obtained using one-way ANOVA tests, while

# *p-value for the gender distribution in the three groups was obtained using a x^2^test. p < 0.05 is considered significant. NC is healthy controls, OCD indicates the patients with obsessive-compulsive disorder, and SZ is Schizophrenia patient. PANSS, Positive and Negative Syndrome Scale. HARS, the Hamilton Anxiety Rating Scale; 24-HDRS, the 24-item Hamilton Rating Scale for Depression; Y-BOCS, the Yale Brown Obsessive Compulsive Scale*.

### Imaging Acquisitions and Preprocessing

Images were acquired with a 3.0-Tesla Siemens Trio Tim with a 12-channel phased array head coil at the Department of Medical Imaging, Wuxi People's Hospital, Nanjing Medical University. All participants have obtained both DTI data and high-resolution T1-weighted axial images. The DTI images were obtained with the following parameters: diffusion was measured along 64 non-collinear directions (b value = 1,000 s/mm^2^), and an additional image without diffusion weighting (i.e., b = 0 s/mm^2^), TR/TE = 7,000 ms/92 ms, flip angle = 90°, field of view (FOV) = 256 × 256 mm^2^, matrix = 128 × 128, slice thickness/gap = 3/0 mm, acquisition voxel size = 2 × 2 × 3 mm^3^. The high-resolution T1-weighted axial images were obtained with the following parameters: repetition time/echo time (TR/TE) = 2530/3.44 ms, thickness/gap = 1.0/0 mm, flip angle = 7°, inversion time = 400 ms, matrix = 256 × 256, FOV = 256 × 256 mm^2^, acquisition voxel size = 1 × 1 × 1 mm^3^.

Image preprocessing was performed using the diffusion toolbox of functional magnetic resonance imaging of the brain (FMRIB) software library (FSL, http://fsl.fmrib.ox.ac.uk/fsl/fslwiki/). The preprocessing steps included eddy current and motion artifact correction, diffusion tensor estimation, and tractography. Corrections for eddy current distortions and head motion were performed by applying a rigid-body transformation of each diffusion-weighted image to the b0 image. Then, the b-matrix of each sample was reoriented to provide a more accurate estimate of tensor orientations. The diffusion tensor matrix was calculated according to the Stejskal and Tanner equation. Three eigenvalues and eigenvectors were obtained by diagonalization of the tensor matrix, and then FA maps were computed. Each b0 image was then registered to Montreal Neurological Institute (MNI) space through the corresponding T1 image by using Diffusionkit (Xie et al., 2016) (https://www.nitrc.org/projects/diffusionkit). The image registration of Diffusionkit is implemented by NiftyReg which is an open-source software for efficient medical image registration and mainly developed by the Centre for Medical Image Computing at University College London. This transform information was saved for later use. The diffusion images remained in native space.

Three-dimensional tract reconstruction was implemented by diffusion toolkit (http://www.trackvis.org). Whole-brain tractography was obtained using the Fiber Assignment by Continuous Tracking (FACT) algorithm (Mori et al., 1999) and the propagation was terminated if either a minimum angle threshold at 50° was violated or a voxel was encountered with FA below 0.2.

### Structural Network Construction

The automated anatomical labeling (AAL) atlas (http://www.gin.cnrs.fr/en/tools/aal-aal2/) (Tzourio-Mazoyer et al., 2002) with 116 regions (Supplementary Material, Table S1) was employed as nodes. Using the inverse of the transform information, the AAL atlas in MNI space was registered into each subject's native space. Edges were defined as inter-regional fibers between each pair of nodes and satisfied the conditions: (1) at least two fibers with two endpoints passed through pair-wise nodes, and (2) the length of the passing fibers were >10 mm. Here, FA value was treated as a network connection's weight. Specifically, each edge's FA weight was calculated by averaging the FA values of all the fibers which constituted this edge, and each fiber's FA value was the mean of the FA values of all voxels in this fiber track. A group threshold was applied to balance the influences of false-positive and false-negative reconstructions of fibers (de Reus and van den Heuvel, 2013). At first, edges that were present in at least 40% of all group members were retained while others were set to zeros in each group. Then, all edges that were present in at least 40% of the entire samples were retained. All subsequent analyses were conducted on this group threshold network.

### Network Measure Analysis

For global network characteristics, we employed network strength and global efficiency. For local network measures, we computed two popular network metrics including nodal strength and nodal efficiency. Their formal math definitions and meanings have been described in Rubinov and Sporns (2010), and we also presented these descriptions in the Supplementary Materials. These measures were calculated on WM network of each subject by using the Brain Connectivity Toolbox (http://www.nitrc.org/projects/bct/) (Rubinov and Sporns, 2010). Due to the age differences among groups, the interaction between age and network metrics within each group was regressed out, respectively. All comparisons involving the network metrics were analyzed using one-way ANOVAs, separately. To address the problem of multiple comparisons in the ANOVA tests, a false discovery rate (FDR) (Benjamini and Hochberg, 1995) correction was implemented with the threshold of *q* = 0.05. The *post-hoc* pair-wise comparisons were then performed using independent *t*-tests. A value of *p* < 0.05 was considered significant.

### NBS Analysis

NBS was proposed by Zalesky et al. (2010), which was a nonparametric method to eliminate the multiple comparison problem encountered when conducting mass univariate significance tests. Statistical significance was detected for specific subsets of nodes that are connected in topological space. Due to the comparisons among three groups, we first used NBS to conduct a one-way ANCOVA analysis and age was as a covariate. The general calculation procedures were as below. First, a primary threshold (*F*-value = 2.2) was applied to an *F*-test, which was calculated for each edge to construct a set of suprathreshold connections. This identified all the possible mutually connected components (or sub-networks) in a WM network at the primary threshold level. Then, the size of the actual remaining sub-network *s* was determined. To estimate the significance of each sub-network, the null distribution of the sub-network size was empirically derived using a nonparametric permutation approach (5,000 permutations). For each permutation, all of the samples were randomly shuffled among the groups, and the *F* statistic was computed independently for each edge. Afterwards, the same threshold was applied to retain edges above this threshold and the maximal sub-network size was restored. Lastly, corrected *p*-value was determined by calculating the proportion of the 5,000 permutations for which the maximal shuffled sub-network was greater than *s*. The *post-hoc* pair-wise comparisons were then performed using independent *t*-tests and also set age as covariate in NBS. The processing steps of independent *t*-tests were similar to those of the above one-way ANCOVA, except the steps of suprathreshold edges establishment in which conducted a *t*-test for each edge rather than F statistic. All the pair-wise group comparisons were conducted 5,000 permutations and set *p* < 0.05 (uncorrected) as thresholds. A value of *p* < 0.05 (corrected) was considered significant results.

### Hub Distribution Analysis

Here, betweenness centrality was used to define a hub node and its formal definition was presented in Supplementary Materials. We applied the Euclidean distance to assess the dissimilarity of hub distributions among the group of SZ, OCD, and NC. Briefly, we first defined an 1 × *N* probability vector for each diagnostic group (*N* = 116 was the total number of nodes). For each diagnostic group, the entry *f*_*i*_ of the probability vector represented the probability of being hub for node *i*, normalized by the number of samples in this group (hence *f*_*i*_ values ranged from 0 to 1). Next, we calculated the Euclidean distance based on these probability values between any two groups. Mathematically, the distance *D* of pair-wise groups was defined as:

(1) $$\begin{array}{l} D=\sqrt{{\overset{N}{\underset{i=1}{\sum }}\left(f_{i}^{G1}-f_{i}^{G2}\right)}^{2}},i=1,\ldots ,N \end{array}$$

where the superscripts G1 and G2 indicated different groups.

## Results

### Difference in Network Measures

Significant group effects on network strength (*F* = 5.61, *p* = 0.005) and global efficiency (*F* = 9.64, *p* < 0.001) were observed in the analyses of the three groups. *Post hoc* analyses revealed a significantly decreased network strength in the SZs compared with NCs and OCDs (*p* = 0.001 for SZs VS. NCs, *p* = 0.021 for SZs VS. OCDs). Global efficiency was significantly decreased in the SZs compared with NCs and OCDs (*p* < 0.001 for SZs VS. NCs, *p* = 0.002 for SZs VS. OCDs).

Significant group effects on nodal strength and nodal efficiency among the three groups were observed in the four frontal regions [right middle frontal gyrus (MFG), right orbital part of middle frontal gyrus (ORBmid), right orbital part of inferior frontal gyrus (ORBinf), and left medial orbital part of superior frontal gyrus (ORBsupmed)], two temporal regions [right fusiform gyrus (FFG) and left heschl gyrus (HES)], and two subcortical nucleis [right thalamus (THA) and right putamen (PUT)] (Table 2 and **Figure 2**). *Post hoc* analyses found most of these regions exhibited a reduced nodal strength and nodal efficiency in SZs compared with OCDs and NCs. Specifically, the SZs displayed significantly lower nodal efficiency in the right ORBinf, right ORBsupmed, right FFG, left HES, right THA and right PUT than OCDs, and NCs. Notably, the right PUT in nodal strength was also reduced in SZs relative to OCDs and NCs. Compared with NCs, the right MFG and ORBmid indicated reduced nodal strength and nodal efficiency in OCDs. Only the right MFG was disrupted in both OCDs and SZs, with a lower nodal strength and nodal efficiency than NCs. All these network metric results were also plotted in a bar figure as shown in the Supplementary Figure S2. Moreover, we have computed the small-worldness characteristics for the three groups (OCD, SZ, and NC), and observed that all groups exist this characteristics, but there was no significant pair-wise group differences among the three groups (*F* = 1.45 and *p* = 0.238 for one-way ANOVA analysis, as shown in the Supplementary Figure S3). Additionally, we also calculated the correlations between network metrics and the clinical scale scores (i.e., Y-BOCS scale and PANSS scale) for OCD and SZ, respectively, and we only found that the sum scores of Y-BOCS is significantly correlated with the right ORBmid in nodal efficiency (*r* = 0.61, *p* = 0.0023), which was shown in the Supplementary Figure S4.

**Table 2:** Specific nodes with significant between-group differences in the network metrics.

**Metric**	**Regions**	***p*-value (corrected) of ANOVA**	***T*-value (*p*-value) of *post hoc* test**		
			**OCD vs. NC**	**SZ vs. NC**	**OCD vs. SZ**
S*_*i*_*	MFG.R	0.045 ^*^	−2.54 (0.006)	−3.43 (<0.001)	NS
S*_*i*_*	ORBmid.R	0.001 ^*^	−4.72 (<0.001)	NS	−4.17 (<0.001)
S*_*i*_*	LING.L	0.019 ^*^	NS	−4.16 (<0.001)	2.41 (0.010)
S*_*i*_*	PUT.R	0.026 ^*^	NS	−3.75 (<0.001)	2.33 (0.012)
S*_*i*_*	Cere8.R	0.041 ^*^	NS	−2.95 (0.002)	3.19 (0.001)
E*_*i*_*	MFG.R	0.034 ^*^	−2.0 (0.025)	−3.70 (<0.001)	NS
E*_*i*_*	ORBmid.R	0.061	−3.0 (0.002)	NS	NS
E*_*i*_*	ORBinf.R	0.028 ^*^	NS	−4.01 (<0.001)	3.30 (<0.001)
E*_*i*_*	ORBsupmed.R	0.034 ^*^	NS	−3.28 (<0.001)	3.2 (0.001)
E*_*i*_*	CAL.L	0.034 ^*^	NS	−3.60 (<0.001)	2.18 (0.017)
E*_*i*_*	LING.L	0.034 ^*^	NS	−3.97 (<0.001)	2.09 (0.021)
E*_*i*_*	FFG.R	0.034 ^*^	NS	−3.55 (<0.001)	2.28 (0.013)
E*_*i*_*	PUT.R	0.034 ^*^	NS	−3.26 (<0.001)	2.54 (0.007)
E*_*i*_*	THA.R	0.041 ^*^	NS	−3.32 (0.003)	1.68 (0.049)
E*_*i*_*	HES.L	0.041 ^*^	NS	−2.93 (0.002)	1.68 (0.049)

*S_i_ was nodal strength. E_i_ was nodal efficiency*.

*NS indicated no significant results*.

* *indicated that the p-value was survived after FDR correction*.

*OCD, Obsessive-Compulsive Disorder; SZ, Schizophrenia; NC, Normal Controls; L, left; R, right*.

*MFG, Middle Frontal Gyrus; ORBmid, orbital part of Middle Frontal Gyrus; LING, Lingual Gyrus; PUT, Putamen; Cere8, Cerebelum_8; ORBinf: orbital part of Inferior Frontal Gyrus; ORBsupmed, medial orbital part of Superior Frontal Gyrus; CAL, Calcarine Fissure and Surrounding Cortex; FFG, Fusiform Gyrus; THA, Thalamus; HES, Heschl Gyrus*.

### Difference in Structural Connectivity Patterns

NBS analysis of structural connectivity found significant differences among the three groups (*p* < 0.001, corrected for multiple comparisons). *Post hoc* comparisons revealed the three significantly different sub-networks between the groups (Table 3 and **Figure 2**). (1) Compared with NCs, OCDs showed significantly fewer connections among frontal-limbic areas (corrected *p* = 0.026) including the bilateral dorsolateral part of Superior Frontal Gyrus (SFGdor) and the right medial part of Superior Frontal Gyrus (SFGmed)], and the left Anterior Cingulate and paracingulate Gyri (ACG). Additionally, (2) SZs had significantly fewer connections in the main cortices and subcortical nuclei than NCs (corrected *p* < 0.001), which involved the occipital regions (i.e., the bilateral CAL, right cuneus, right SOG, right LING, and right inferior occipital gyrus), parietal regions (i.e., the right superior parietal gyrus, inferior parietal gyrus, and postcentral gyrus), temporal regions (i.e., the right FFG), limbic system (i.e., the right parahippocampal gyrus), and basal ganglia (i.e., the right PUT and pallidum). Interestingly, (3) OCDs displayed significantly more connections between the basal ganglia (i.e., PUT and pallidum) and visual/auditory cortices (i.e., cuneus and postcentral gyrus) than SZs (corrected *p* < 0.001).

**Table 3:** Sub-network with significant between-group difference based on *post-hoc* of NBS analysis.

**Network edges**	**Network**	***t-* and *p*-Value**
**OCD vs. NC**		
SFGdor.R–SFGdor.L	ECN–ECN	*t* = 2.29, *p* < 0.05
SFGdor.R–ACG.L	ECN–Limbic	*t* = 2.22, *p* < 0.05
SFGmed.R - ACG.L	ECN–Limbic	*t* = 1.95, *p* < 0.05
**SZ vs. NC**		
CAL.L–CAL.R	VN–VN	*t* = 3.22, *p* < 0.005
CAL.L–CUN.R	VN -VN	*t* = 2.30, *p* < 0.025
CUN.R–SOG.L	VN–VN	*t* = 2.41, *p* < 0.005
PHG.R–FFG.R	Limbic–DAN	*t* = 2.99, *p* < 0.005
CAL.R–FFG.R	VN–DAN	*t* = 2.20, *p* < 0.025
IOG.R–FFG.R	VN–DAN	*t* = 2.82, *p* < 0.005
SPG.R–IPL.R	DAN–ECN	*t* = 2.29, *p* < 0.025
ROL.R–PUT.R	AN–BGN	*t* = 5.88, *p* < 0.005
CUN.R–PUT.R	VN–BGN	*t* = 5.85, *p* < 0.005
LING.R–PUT.R	DMN–BGN	*t* = 2.31, *p* < 0.025
SPG.R–PUT.R	DAN–BGN	*t* = 3.06, *p* < 0.005
PoCG.R–PAL.R	SMN–BGN	*t* = 2.50, *p* < 0.005
SPG.R–PAL.R	DAN–BGN	*t* = 2.21, *p* < 0.025
**OCD vs. SZ**		
ROL.R–PUT.R	AN–BGN	*t* = 5.09, *p* < 0.005
CUN.R–PUT.R	VN–BGN	*t* = 4.29, *p* < 0.005
PoCG.R–PUT.R	SMN–BGN	*t* = 2.29, *p* < 0.025
PoCG.R–PAL.R	SMN–BGN	*t* = 2.39, *p* < 0.025

*SFGdor, dorsolateral part of Superior Frontal Gyrus; SFGmed, medial part of Superior Frontal Gyrus; ACG, Anterior Cingulate and Paracingulate Gyri; SOG, Superior Occipital Gyrus; TPOsup, Temporal Pole part of Superior temporal gyrus; CUN, Cuneus; PHG, Parahippocampal Gyrus; IOG, Inferior Occipital Gyrus; SPG, Superior Parietal Gyrus; IPL, Inferior Parietal (but supramarginal and angular gyri); ROL, Rolandic Operculum; PoCG, Postcentral Gyrus; PAL, Pallidum*.

### The Dissimilarity of Hub Distribution

**Figure 1:**
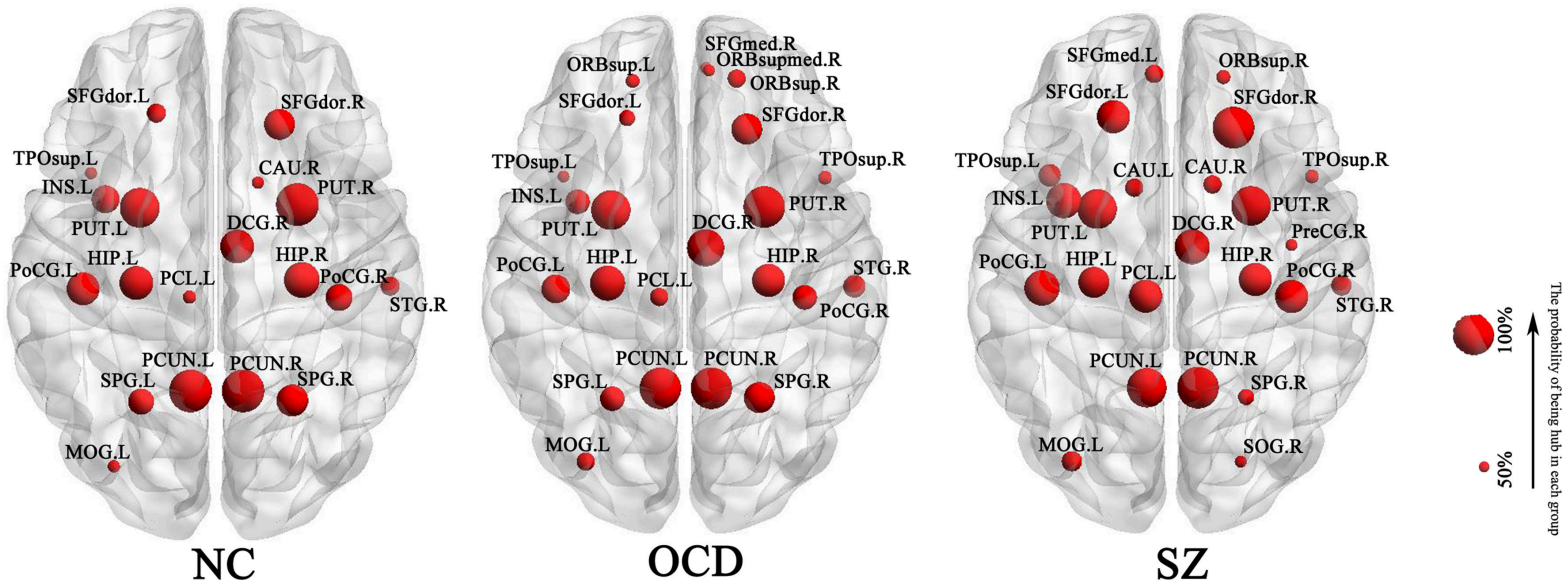
Hub distribution of each group. It was shown the hub whose probability of being hub was >50%. OCD, Obsessive-Compulsive Disorder; SZ, Schizophrenia; NC, Normal Controls. L, left; R, right. SFGdor, dorsolateral part of Superior Frontal Gyrus; TPOsup, Temporal Pole part of superior temporal gyrus; INS, Insula; PUT, Putamen; HIP, Hippocampus; PoCG, Postcentral Gyrus; PCL, Paracentral Lobule; PCUN, Precuneus; SPG, Superior Parietal Gyrus; MOG, Middle Occipital Gyrus; CAU, Caudate nucleus; DCG, Median cingulate and paracingulate gyri; STG, Superior Temporal Gyrus; ORBsup, orbital part of Superior Frontal Gyrus; SFGmed, medial part of Superior Frontal Gyrus; ORBsupmed, medial orbital part of Superior Frontal Gyrus; PreCG, Precental Gyrus; SOG, Superior Occipital Gyrus.

A node was identified as hub if its probability of being hub in a group was larger than 50%. Nineteen, 23 and 24 hubs were determined for NC, OCD, and SZ group, respectively (Figure 1). Euclidean distance was used to assess the dissimilarity of hub distribution between the groups. The higher Euclidean distance was, the more dissimilarity between groups had. As a result, the Euclidean distance was 0.38 between OCDs and NCs, 0.54 between SZs and NCs, and 0.58 between OCDs and SZs, suggesting that OCDs and NCs had the most similar hub distribution, while OCDs and SZs had more disparity.

## Discussion

**Figure 2:**
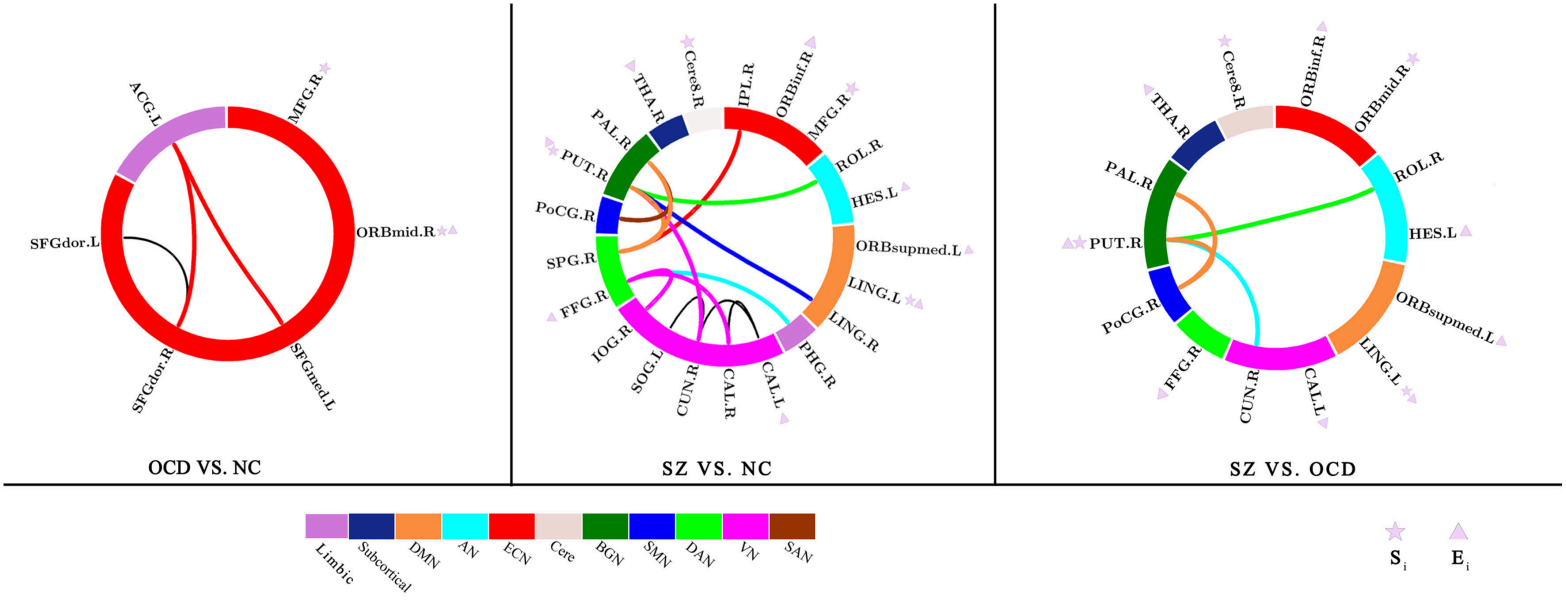
Summarizing network characteristics and network-based statistic results. All nodes were assigned to different functional networks. ECN, Executive Control Network; BGN, Basal Ganglia Network; SAN, Salient Network; VN, Visual Network; DAN, Dorsal Attention Network; AN, Auditory Network; DMN, Default Mode Network; SMN, Sensorimotor Network. S_*i*_, Nodal strength; E_*i*_, Nodal efficiency. MFG, Middle Frontal Gyrus; ORBmid, orbital part of Middle Frontal Gyrus; LING, Lingual Gyrus; Cere8, Cerebelum_8; ORBinf, orbital part of Inferior Frontal Gyrus; CAL, Calcarine Fissure and Surrounding Cortex; FFG, Fusiform Gyrus; THA, Thalamus; HES, Heschl Gyrus; CUN, Cuneus; PHG, Parahippocampal Gyrus; IOG, Inferior Occipital Gyrus; IPL, Inferior Parietal (but supramarginal and angular gyri); ROL, Rolandic Operculum; PAL, Pallidum.

This work is the first attempt to directly compare the topological alterations of WM networks in drug-naive patients with SZ and OCD as well as NC. Three primary findings were as below: (1) for global network characteristics, as indicated by reduced network characteristics, the organization of the WM networks was significantly disrupted with a distinct abnormal pattern in each disease, and more abnormalities were located in SZs than OCDs. Moreover, as indicated by dissimilarity of hub distribution, OCDs, and NCs had the most similar hub distribution, while OCDs and SZs showed more disparity; (2) the SZs displayed significantly lower nodal efficiency or nodal strength in the PUT, THA, and OFC than OCDs; (3) the SZs displayed significantly less connections between the basal ganglia and visual/auditory cortices than OCDs. Figure 2 summarized the nodal metrics and NBS results.

### Disrupted Global Topological Organization in WM Networks

Network strength and global efficiency significantly decreased in the SZs compared with NCs and OCDs, but they were no significant difference between OCDs and NCs. These findings are largely consistent with previously observed network alterations in patients with SZ (Zalesky et al., 2011; van den Heuvel and Fornito, 2014; Fornito and Bullmore, 2015). Only very few studies have examined alterations in structural networks in OCD using graph theory methods. A clear and consistent result regarding whether network-level measures exhibit significant differences between OCD and NC is still lacking, as discrepancies have been observed. Kim et al. (2013) focused on cortical thickness and reported no clear distinction between OCD and NC in terms of network-level efficiency measures (i.e., global efficiency). Zhong et al. (2014) defining structural networks based on diffusion data, reported decreased global efficiency in OCD. In contrast, (Reess et al., 2016) only found OCD patients showed a trend for a reduced global degree strength and total fiber count, which did not reach a significant level. These differing effects may be due to the limited statistical power of studies examining a small sample and the differences in the characteristics of recruited patients (i.e., disease severity level and cultural background) or research strategies utilized (i.e., imaging protocols) across studies. Moreover, previous studies also reported that SZ represented a more severe biological disturbance with greater neurological abnormalities than OCD (Kim et al., 2003; Ha et al., 2005; Riffkin et al., 2005). Lower network strength was associated with the sparse connectivity of networks, whereas decreasing global efficiency reflects an altered global integration of WM networks and is majorly related to long-range connections. This finding implies a gradient in the extent of alterations such that SZ > OCD, which suggests SZ has more serious damage to the efficiency of global information interaction across the whole WM network, while OCD has a relatively intact network organization.

In addition, the hub distribution analyses revealed that OCD and NC had the most similar hub distribution, while those between OCD and SZ were more disparity. Hub architecture serves as a foundational backbone supporting communication among functionally specialized networks (van den Heuvel et al., 2012). These dissimilarities of both disorders suggest they have distinct hub distribution patterns, which implicates the different information interaction processes in the pathological statuses.

### Disorder-Related Distinctions of Regional Characteristics in WM Networks

Findings from the regional characteristics identified a decrease of nodal efficiency in SZs relative to both OCDs and NCs involving a wide range of regions (i.e., the right PUT, THA, ORBinf, ORBsupmed, FFG, and the left HES). In SZ, these regions have exhibited abnormalities in a broad range of studies (Konrad and Winterer, 2008; Zalesky et al., 2011; Wang et al., 2012; Zhang et al., 2012). Moreover, OCD was mainly showed a reduction of nodal efficiency in the frontal regions (i.e., right MFG and ORBmid). Such abnormalities have been reported in prior OCD studies (Zhong et al., 2014). The classical neurobiological models of OCD suggest that the disturbance of cortico-striato-thalamo-cortical (CSTC) circuits (i.e., OFC, ACG, and striatum) play a crucial role in the pathophysiological mechanisms underlying OCD (Menzies et al., 2008; Harrison et al., 2013; Piras et al., 2013). Unlike OCD, it is hard for SZ to summarize its abnormities into a circuit. Consistent studies reported widespread alterations of regional morphology, volume, WM integrity, and network properties in the thalamus, frontal, temporal, and parietal cortices in SZ patients (van den Heuvel and Fornito, 2014; Wheeler and Voineskos, 2014; Fornito and Bullmore, 2015). Our findings are in line with the prior studies. Nodal efficiency quantifies the importance of a node for the communication within a network (Rubinov and Sporns, 2010). An aberrant nodal efficiency reflects abnormalities in inter-regional connectivity. These alterations indicate that SZ is widely disrupted in inter-regional connectivity and affects the efficiency of information communication across the whole network, while OCD mainly altered in the frontal regions. Additionally, most of these altered regions have significant differences between these two disorders, which may be a valuable marker for distinguishing them. Thus, WM network analyses have sufficient sensitivity to identify the distinctions between SZ and OCD.

This study also found a significant decrease in nodal strength and efficiency common to both SZ and OCD groups in the right MFG. The abnormal right MFG has been reported in prior WM network studies of SZ (Wang et al., 2012) and OCD (Zhong et al., 2014). The MFG plays a vital role in executive control, attention, and working memory, which is involved in the pathogenesis of SZ (Kikinis et al., 2010; Fornito et al., 2012; Quan et al., 2013) and OCD (Muller and Roberts, 2005; Nakao et al., 2009; Snyder et al., 2015). Nodal strength provides a simple measure of direct interaction. Thus, a reduced nodal strength and efficiency of the right MFG implies an abnormal information transfer of this region in both disorders, which may contribute to the common symptoms of these disorders to some extent.

### Disorder-Related Distinctions of Sub-networks in WM Networks

As depicted in Figure 2 and Table 3, it was shown different abnormalities when comparing the two disorders with the NCs individually. The number of impaired sub-network in OCD was smaller than those of SZ. The abnormalities of OCD are located within the frontal–limbic system, while SZ predominately pertains to BGN, dorsal attention network (DAN), and visual network (VN). These disrupted network connections are most documented in many previous studies of SZ (Jiang et al., 2013; Cordon et al., 2015; Tu et al., 2015; Jimenez et al., 2016) and OCD (Piras et al., 2013; Goettlich et al., 2014; Reess et al., 2016). In addition, the connections of BGN–visual/auditory cortices were the major differences of both disorders. More wide-range decrease in functional network connections involving sensory and subcortical regions have been observed in patients with SZ (Kaufmann et al., 2015; Skåtun et al., 2016). Altered sensory processing provide inaccurate input to higher order regions (i.e., frontal regions), which may result in maladaptive activities and adaptations in neural circuits. Then, these maladaptations may feed back into sensory processing circuits and produce a loop for persistent disturbances within the brain network, which may lead to the clinical manifestations observed in SZs. This finding suggests that the clinical symptoms of SZ and OCD may underlie different biological bases in the brains.

Notably, in this study, whatever using which analyses methods, the right PUT consistently exhibited significant decrease in nodal strength/efficiency and more sparse connections in the SZs compared with OCDs. Moreover, these decreases also displayed in SZs relative to NCs, but they do not appear in OCDs. The PUT has a close association with the pathological mechanism of SZ (Wang et al., 2012). In fact, when investigating the involvement of putamen in SZ, the key findings are to do with the dopamine system, the symptoms and the site of antipsychotic drug action (Hall et al., 1994; Dazzan et al., 2005; Farid and Mahadun, 2009). Moreover, the gray matter volume of putamen showed a potential to be a transdiagnostic marker of vulnerability to psychopathology including of SZ and OCD (Gong et al., 2018). Our data implicates that the WM abnormality of right PUT may aggravate the burden on the information transform efficiency in SZ, but this region is relatively intact in OCD. The way of information interaction in the right PUT are different between SZs and OCDs, and the former has a serious abnormality. Taken collectively, these evidences highlight the importance of the PUT in the understanding of pathophysiology of SZ and OCD.

### Limitations and Conclusions

There may be potential heterogeneity in current patient cohort, like symptom-based subgroup taxonomy for OCD (Calamari et al., 2004). To identify potential subtypes of OCD, it requires special research strategy and data for a large cohort of patients in the future study. In case of SZ, use of traditional subtypes is now uncommon in the scientific literature (Braff et al., 2013). It is noteworthy that these patients are drug-naive participants, who were unaffected by either psychotherapy or psychopharmacotherapy. A prior study reported that the use of antipsychotic drugs in SZ patients was related to the occurrence of an obsessive-compulsive symptom (Schirmbeck and Zink, 2013). Hence, this confounding factor was excluded in this study. We also note that the current study does not completely age-matched between two patient groups although most of their age range from 18 to 45 years. Therefore, we conducted an additional age-matched analysis using subsets of patients (see validation of age-matched samples in the Supplementary Materials) and found that the main results have a good reproducibility, suggesting that the findings are robust and reliable, and the age has little effect on our main results. Finally, it certainly requires more experimental evidence to support the clinical application of our findings. And one of the first considerations is the reliability of our used measurements. In clinical practice, the reliability of any tool and measurement should reach at least larger than 0.8 (Xing and Zuo, 2018). However, we cannot directly assess the reliability of our used network metrics because of the only one time scanning DTI image of each volunteer. But the prior relevant DTI network studies suggested that this kind of network and its popular network metrics such as nodal strength had a substantial reliability (Buchanan et al., 2014), especially Yuan et al. (2018) reported that FA weights were more suited for DTI connectome studies in adolescents. We will take into account to include the reliability of employed measures in our future work.

In summary, this study investigates the association of SZ and OCD in the perspective of the topological organization of WM networks under the same research framework. It was found that these two disorders have the different level of anatomical impairment and some distinct topological patterns. As for the impairment levels, SZ is more serious than OCD. Regarding the deficit patterns, the alterations of OCD predominately pertain to the frontal regions (i.e., OFC and MFG). But SZ exhibits a wide range of abnormal patterns involving main cortices (i.e., the frontal, parietal, occipital, and temporal region) and subcortical nuclei (i.e., striatum and THA). Moreover, the nodal efficiency of the frontal and temporal regions, as well as striatum can reflect the differences in the two disorders, which may be a valuable marker for distinguishing them, especially to the PUT which may be closely related to these disorders. It is our aim that this information will improve and add value to further research to determine the nature of OCD and SZ.

## Author Contributions

LT designed the study and wrote the protocol. JQ and JS managed the data analyses. SW, FZ, and ZZ provided the data necessary for our analysis. HN analyzed the results. JQ, JS, and LT wrote the manuscript. All authors contributed to and have approved the final manuscript.

### Conflict of Interest Statement

The authors declare that the research was conducted in the absence of any commercial or financial relationships that could be construed as a potential conflict of interest.
