# Supplementary material for: The Shared and Distinct White Matter Networks Between Drug-Naive Patients With Obsessive-Compulsive Disorder and Schizophrenia
Source: Front Neurosci. 2019 Feb 21;13:96. doi: 10.3389/fnins.2019.00096 (PMC6393388; doi:10.3389/fnins.2019.00096)
Supplement: Supplementary file 1 [file Data_Sheet_1.docx]

**Supplementary materials**

**Table S1.** Cortical regions of interest defined in the current study

| **Index** | **Regions** | **Abbreviation** | **Index** | **Regions** | **Abbreviation** |
| --- | --- | --- | --- | --- | --- |
| 1,2 | Precental gyrus | PreCG | 63,64 | Supramarginal gyrus | SMG |
| 3,4 | Superior frontal gyrus, dorsolateral | SFGdor | 65,66 | Angular gyrus | ANG |
| 5,6 | Superior frontal gyrus, orbital part | ORBsup | 67,68 | Precuneus | PCUN |
| 7,8 | Middle frontal gyrus | MFG | 69,70 | Paracentral lobule | PCL |
| 9, 10 | Middle frontal gyrus, orbital part | ORBmid | 71,72 | Caudate nucleus | CAU |
| 11,12 | Inferior frontal gyrus, opercular part | IFGoperc | 73,74 | Lenticular nucleus, putamen | PUT |
| 13,14 | Inferior frontal gyrus, triangular part | IFGtriang | 75,76 | Lenticular nucleus, pallidum | PAL |
| 15,16 | Inferior frontal gyrus, orbital part | ORBinf | 77,78 | Thalamus | THA |
| 17,18 | Rolandic operculum | ROL | 79,80 | Heschl gyrus | HES |
| 19,20 | Supplementary motor area | SMA | 81,82 | Superior temporal gyrus | STG |
| 21,22 | Olfactory cortex | OLF | 83,84 | Temporal pole: STG | TPOsup |
| 23,24 | Superior frontal gyrus, medial | SFGmed | 85,86 | Middle temporal gyrus | MTG |
| 25,26 | Superior frontal gyrus, medial orbital | ORBsupmed | 87,88 | Temporal pole: MTG | TPOmid |
| 27,28 | Gyrus rectus | REC | 89,90 | Inferior temporal gyrus | ITG |
| 29,30 | Insula | INS | 91,92 | Cerebelum_Crus1 | Crus1 |
| 31,32 | Anterior cingulate and paracingulate gyri | ACG | 93,94 | Cerebelum_Crus2 | Crus2 |
| 33,34 | Median cingulate and paracingulate gyri | DCG | 95,96 | Cerebelum_3 | Cere3 |
| 35,36 | Posterior cingulate gyrus | PCG | 97,98 | Cerebelum_4_5 | Cere4_5 |
| 37,38 | Hippocampus | HIP | 99,100 | Cerebelum_6 | Cere6 |
| 39,40 | Parahippocampal gyrus | PHG | 101,102 | Cerebelum_7b | Cere7b |
| 41,42 | Amygdala | AMYG | 103,104 | Cerebelum_8 | Cere8 |
| 43,44 | Calcarine fissure and surrounding cortex | CAL | 105,106 | Cerebelum_9 | Cere9 |
| 45,46 | cuneus | CUN | 107,108 | Cerebelum_10 | Cere10 |
| 47,48 | Lingual gyrus | LING | 109 | Vermis_1_2 | VM1_2 |
| 49,50 | Superior occipital gyrus | SOG | 110 | Vermis_3 | VM3 |
| 51,52 | Middle occipital gyrus | MOG | 111 | Vermis_4_5 | VM4_5 |
| 53,54 | Inferior occipital gyrus | IOG | 112 | Vermis_6 | VM6 |
| 55,56 | Fusiform gyrus | FFG | 113 | Vermis_7 | VM7 |
| 57,58 | Postcentral gyrus | PoCG | 114 | Vermis_8 | VM8 |
| 59,60 | Superior parietal gyrus | SPG | 115 | Vermis_9 | VM9 |
| 61,62 | Inferior parietal, but supramarginal and angular gyri | IPL | 116 | Vermis_10 | VM10 |

Note: The regions are listed based on the template obtained from the AAL atlas.

**Network measure analysis**

To describe the topological organization of white matter structural networks, two graph measures were included here, as below: nodal strength and nodal efficiency. We used betweenness centrality to define hubs and this metric was introduced here. Regarding a review on the uses and interpretations of the measures, please see (Rubinov and Sporns 2010) and the following characterizations.

***Nodal strength***

In a weighted graph G with n nodes, the nodal strength s*_i_* for node *i* was defined as the sum of the weights of direct connecitons of node *i*:

$$s_{i}=\sum_{j\in N} W_{ij}$$

[1]

where N was the set of all nodes in the graph G, and W*ij* was the weight between node *i* and node *j* in the graph. Obviously, the network strength S of network G was computed as the average of s*_i_* for all nodes within set N. Formmaly:

$S=\frac{1}{n}\sum_{i\in N} s_{i}$ [2]

where n was the number of nodes.

***Nodal efficiency***

In a weighted graph G with n nodes, the nodal efficiency e*i* of a node *i* was calculated as the mean of the inverse of the distances of all nodes that directly connected node *i* except the node per se. Formally:

$e_{i}=\frac{1}{n-1}\sum_{j\in,j\neq i} \frac{1}{d_{ij}}$ [3]

where d*ij* was the shortest path length between node *i* and *j* in G, and N was the set of all nodes in the graph. This metric quantified the importance of the nodes for communication within the network. In turn, the global efficiency of a network G with *n* nodes was calculated as:

$E_{glob}(G)=\frac{1}{n}\sum_{i\in N} e_{i}$ [4]

***Betweenness centrality and hub definition***

In a weighted graph G with N nodes, the betweenness centrality b*i* of a node *i* represented a central node that played a key role in control over the information transfer within the G, which was computed as the fraction of shortest paths that passed through node *i* between other nodes. Formally:

$b_{i}=\frac{1}{(n-1)(n-2)}\sum_{h,j\in N,h\neq i,j,i\neq j} \frac{sp_{hj}(i)}{sp_{hj}}$ [6]

where sp*_hj_*(*i*) was the number of shortest path between node *h* and node *j* passing through node *i*, and N was the set of all nodes in the G.

We used betweenness centrality metric to define the hub nodes as follows. For identification of hubs in WM networks, we computed the normalized betweenness centrality as $h_{i}=b_{i}/\left\langle b_{i}\right\rangle$, where $\left\langle b_{i}\right\rangle$ was the mean nodal betweenness centrality of each FA-weighted WM network. A node was classified as a hub, depending on whether its $h_{i}$ value was 1.5 times larger than the $\left\langle b_{i}\right\rangle$ of this network. Thus, each WM network had defined its own hubs.

**Validation of age-matched samples**

We note that this study does not completely age-matched between two patient groups, although most of their ages range from 18 to 45 years. Therefore, we conducted an additional age-matched analysis using subsets of patients and their detailed demographic information was shown as below (supplementary table S2). We validated the main results from the following aspects including network characteristics, structural connectivity patterns and dissimilarity of hub distribution. During these calculations, the involving parameters are set as the same of the main body.

**Table S2**. Demographic information of age-matched validation samples

| Variables | NC (n=64) | OCD (n=24) | SZ (n=28) | *p* value |
| --- | --- | --- | --- | --- |
| Age (years) | 18-50 (32.59±10.64) | 18-43 (28.83±7.06) | 16-51 (31.75±9.00) | 0.267 |
| Education (years) | 6-23 (14.00±3.68) | 6-19 (13.88±3.04) | 0-19 (11.00±4.56) | 0.002^*^ |
| Gender (M/F) | 40/24 | 18/6 | 17/11 | 0.527^#^ |
| Handedness (R/L/A) | 64/-/- | 24/-/- | 28/-/- | >0.999 |

*Difference in network measures*

Significant group effect on network strength (F=5.19, *p* = 0.007) was observed in the analyses of the 3 groups. Post hoc analyses revealed a significantly decreased network strength in SZs compared with NCs and OCDs (*p* = 0.001 for SZs VS. NCs, *p* = 0.033 for SZs VS. OCDs). However, this time we not find the significant difference in global efficiency. But we found SZs with a decrease trend in normalized global efficiency compared with NCs and OCDs (F = 2.99, *p* = 0.055), where normalized global efficiency was computed as following: at first we produced the 100 random networks for each sample's network and calculated their global efficiency values and averaged these random global efficiency values, then the real global efficiency divided by its corresponding random values got normalized global efficiency.

Significant group effects on nodal strength and nodal efficiency among the 3 groups are presented in the table S3, where the overlap column with a tick means that the results of validation and main body are same. In the results of nodal strength, both two times are consistent except the right of MFG and Cere8 without significant level. In addition, the results of nodal efficiency in both times showed a good overlap except the right ORBsupmed region which had no significant difference but its left reached the significant level.

**Table S3.** Specific nodes with significant between-group differences in the network metrics

| **Metric** | **Overlap** | **Regions** | *p* **value (corrected) of ANOVA** | **T value (** *p* **value) of post hoc test** | | |
| --- | --- | --- | --- | --- | --- | --- |
|  |  |  |  |  |  |  |
|  |  |  |  | **OCD vs. NC** | **SZ vs. NC** | **OCD vs. SZ** |
| S*_i_* | √ | ORBmid.R | <0.001* | -4.85(<0.001) | NS | -4.27 (<0.001) |
| S*_i_* | √ | LING.L | 0.034* | NS | -3.91 (<0.001) | 1.84 (0.036) |
| S*_i_* | √ | PUT.R | 0.024* | NS | -3.88 (<0.001) | 2.51 (0.008) |
| S*_i_* | √ | MFG.R | 0.055 | -2.13(0.018) | -3.57 (<0.001) | NS |
| S*_i_* | √ | Cere8.R | 0.055 | NS | -2.96 (0.002) | 2.93 (0.003) |
| E*_i_* |  | ORBsup.R | 0.028* | NS | -3.47 (<0.001) | 2.29 (0.013) |
| E*_i_* | √ | MFG.R | 0.017* | -2.12 (0.018) | -4.06 (<0.001) | NS |
| E*_i_* | √ | ORBmid.R | 0.017* | -3.66(<0.001) | -2.09 (0.020) | -1.75 (0.044) |
| E*_i_* | √ | ORBinf.R | 0.017* | NS | -3.46 (<0.001) | 2.84 (0.003) |
| E*_i_* |  | ROL.L | 0.038* | NS | -3.46 (<0.001) | 1.72 (0.046) |
| E*_i_* |  | ORBsupmed.L | 0.028* | NS | -3.51 (<0.001) | 2.77 (0.004) |
| E*_i_* |  | REC.R | 0.039* | NS | -3.37 (<0.001) | 2.06 (0.022) |
| E*_i_* |  | INS.L | 0.028* | NS | -3.37 (<0.001) | 2.82 (0.003) |
| E*_i_* |  | INS.R | 0.039* | NS | -3.27 (<0.001) | 1.71 (0.047) |
| E*_i_* |  | DCG.L | 0.048* | NS | -2.86 (0.003) | 2.54 (0.007) |
| E*_i_* | √ | CAL.L | 0.028* | NS | -3.55 (<0.001) | 2.11 (0.020) |
| E*_i_* |  | CUN.R | 0.038* | NS | -3.25 (<0.001) | NS |
| E*_i_* | √ | LING.L | 0.028* | NS | -3.56 (<0.001) | NS |
| E*_i_* | √ | FFG.R | 0.028* | NS | -3.55 (<0.001) | 2.06 (0.022) |
| E*_i_* | √ | PUT.R | 0.017* | NS | -3.73 (<0.001) | 2.70 (0.005) |
| E*_i_* |  | PAL.R | 0.036* | NS | -3.29 (<0.001) | 1.94 (0.029) |
| E*_i_* | √ | THA.R | 0.028* | NS | -3.42 (<0.001) | NS |
| E*_i_* | √ | HES.L | 0.028* | NS | -2.88 (0.003) | 2.38 (0.011) |
| E*_i_* |  | HIP.R | 0.059 | NS | -3.11 (0.001) | NS |

*Differences in structural connectivity patterns*

NBS analysis of structural connectivity found significant differences among the 3 groups (*p*<0.001, corrected for multiple comparisons). Post hoc comparisons revealed the three significantly different sub-networks between the groups (Compared with NC, OCD showed fewer connections among ECN and limbic, corrected *p* = 0.02. Compared with NC, SZ depicted fewer connections in the mian cortices and subcortical nuclei, corrected *p* < 0.001. Compared with OCD, SZ displayed fewer connections between the basal ganglia and visual/auditory cortices, corrected *p* < 0.001), and these distinct connections are shown in the table S4. Compared with the results of main body, this time the sub-network results of SZ-NC and OCD-SZ are replicated well, but OCD-NC has a poor reproducibility. NBS method is good at founding a sub-network. We check the NBS results with one-way ANOVA analysis and found that main body result of OCD - NC (i.e., SFGdor.R - SFGdor.L and SFGdor.R - ACG.L) are also significant edges in the replication results, but those edges have lower significant level than those of replication results (i.e., MFG.R - ORBmid.R and ORBmid.R - TPOmid.R). We speculate that the connections invloving MFG.R - ORBmid.R have priority in this comparison and thus the connections (i.e., SFGdor.R - SFGdor.L and SFGdor.R - ACG.L) are not shown in the post hoc analysis when comparing OCDs to NCs.

**Table S4.** Sub-network with significant between-group difference based on post-hoc of NBS analysis

| **Network edges** | **Network** | *t* **and** *p* **value** |
| --- | --- | --- |
| **OCD vs. NC** |  |  |
| MFG.R - ORBmid.R | ECN – Limbic | *t* =3.14, *p* < 0.05 |
| ORBmid.R - TPOmid.R | Limbic – Limbic | *t* =5.13, *p* < 0.05 |
| **SZ vs. NC** |  |  |
| CAL.L - CAL.R | VN - VN | *t* =3.30, *p* < 0.005 |
| CAL.L - CUN.R | VN -VN | *t* =2.36, *p* < 0.025 |
| CUN.R - SOG..L | VN - VN | *t* =2.42, *p* < 0.005 |
| SOG.L - SOG.R | VN - VN | *t* =2.13, *p* < 0.005 |
| SPG.R - IPL.R | DAN - ECN | *t* =2.24, *p* < 0.025 |
| ROL.R - PUT.R | AN - BGN | *t* =5.88, *p* < 0.005 |
| CUN.R - PUT.R | VN - BGN | *t* =5.88, *p* < 0.005 |
| SPG.R - PUT.R | DAN - BGN | *t* =3.06, *p* < 0.005 |
| PoCG.R - PAL.R | SMN - BGN | *t* =2.59, *p* < 0.005 |
| SPG.R - PAL.R | DAN - BGN | *t* =2.25, *p* < 0.025 |
| **OCD vs. SZ** |  |  |
| ROL.R - PUT.R | AN - BGN | *t* =5.62, *p* < 0.005 |
| CUN.R - PUT.R | VN - BGN | *t* =4.76, *p* < 0.005 |
| SPG.R - PUT.R | DAN - BGN | *t* =2.60, *p* < 0.025 |

*The dissimilarity of hub distribution*

Twenty-seven hubs were defined for NC, OCD and SZ group, respectively (figure S1). The Euclidean distance was 0.68 between OCDs and NCs, 0.72 between SZs and NCs, and 0.83 between OCDs and SZs, which showed the same trend as the results in main body. OCDs and NCs had the most similar hub distribution, while OCDs and SZs had more disparity. Moreover, the overlap of hub distribution between replication results and main body results were 0.83 for OCDs, 0.96 for SZs and 1 for NCs, and we used the formula 7 to calculate these overlap. These overlap values imply that the results of the two times are consistent in a large extent.

$Overlap=\frac{Group_{replication}\cap Group_{mainbody}}{Group_{mainbody}}$ [7]


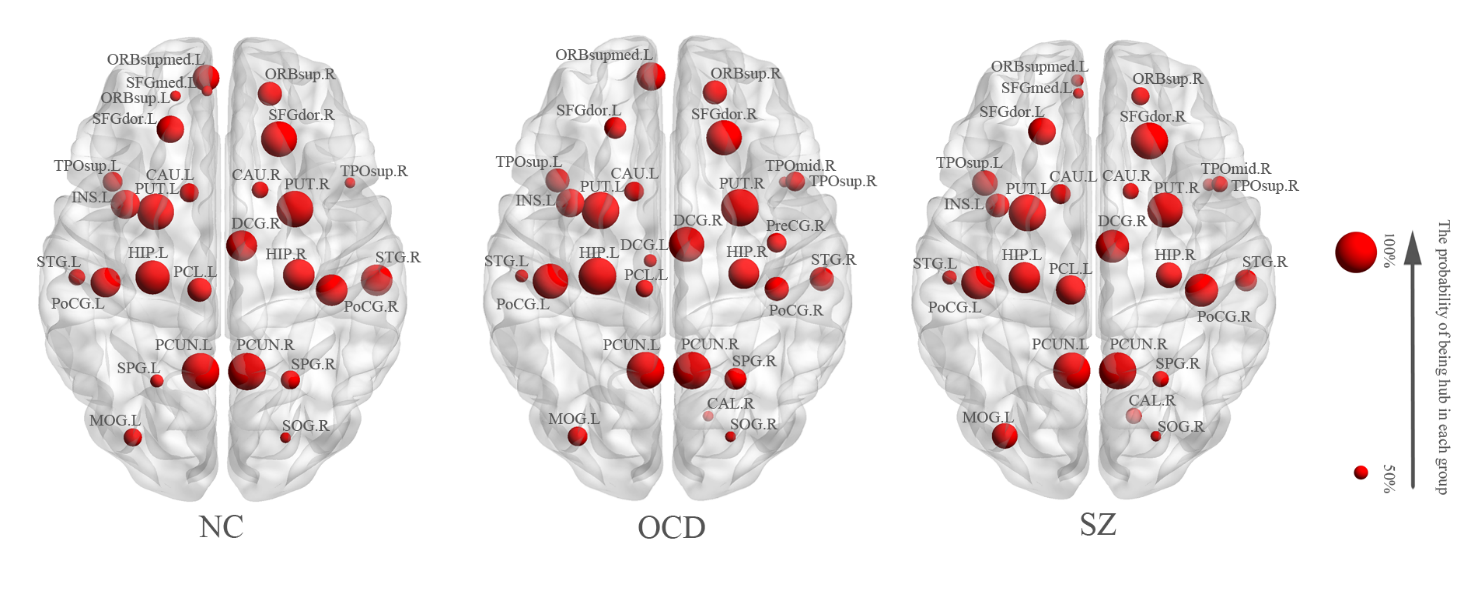
 Figure S1. Hub distribution of NC, OCD and SZ group

In summary, compared with the results of main body, the replication results have a good reproducibility, suggesting that our main findings are robust and reliable, and the age has little effect on our main results.


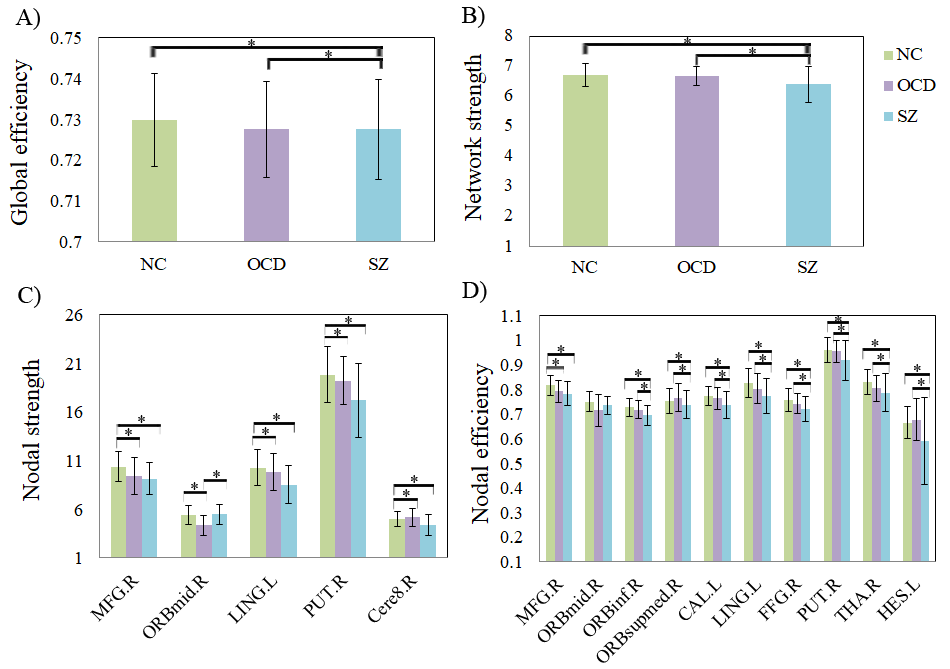


Supplementary Figure S2: The bar figures of network characteristics showing significant between-group differences among the three groups of OCD, SZ and NC.


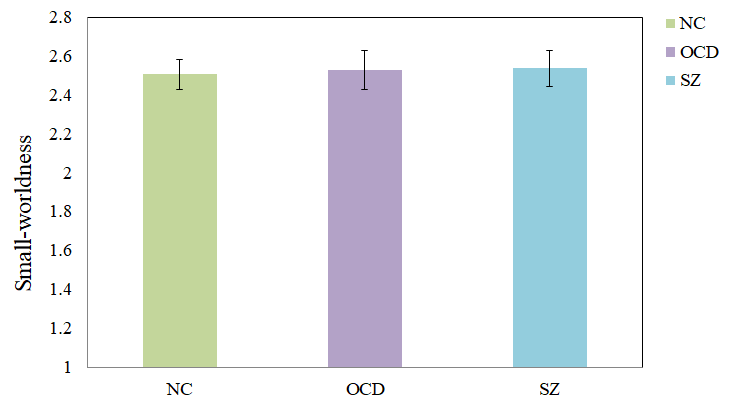


Supplementary Figure S3: The bar figure of small-worldness in the group of OCD, SZ and NC.


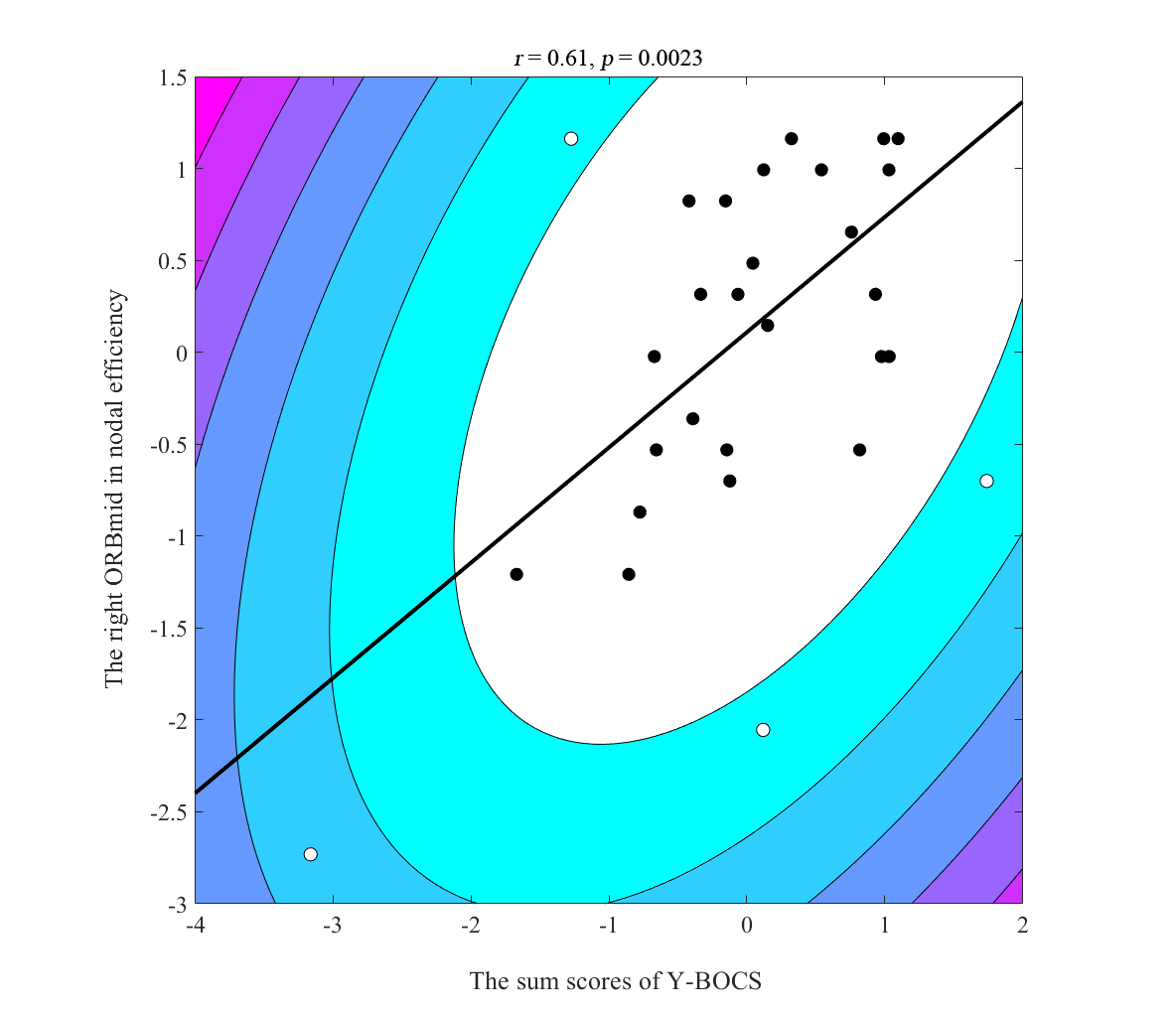


Supplementary Figure S4: The significant correlation result between the sum scores of Y-BOCS and the right ORBmid in nodal efficiency of the group OCD and NC.

**References:**

Rubinov M, Sporns O (2010): Complex network measures of brain connectivity: Uses and interpretations. *NEUROIMAGE* 52:1059-1069.
